# The value of abPG-SGA in the nutritional risk screening of patients with malignant tumors

**DOI:** 10.1097/MD.0000000000038402

**Published:** 2024-05-31

**Authors:** Xiaoling Zhang, Ying Zhang, Yunyi Du, Qian Wu, Xiaoyu Wu, Wenqing Hu, Liang Zong, Xurong Li, Jun Zhao

**Affiliations:** a Department of Oncology, Changzhi People’s Hospital Affiliated to Changzhi Medical College, Changzhi, Shanxi, China; b Department of Respiratory, Pengzhou People’s Hospital, Chengdu, Sichuan, China; c Department of Gastrointestinal Surgery, Changzhi People’s Hospital Affiliated to Changzhi Medical College, Changzhi, Shanxi, China.

**Keywords:** abPG-SGA, hospital patient, malignant tumors, NRS2002, nutritional risk screening

## Abstract

Nutritional risk screening 2002 (NRS2002) is a commonly used tool for screening the risk of malnutrition in hospitalized patients, while patient-generated subjective global assessment (PG-SGA) is a nutritional assessment tool for malignant tumor patients. However, there are still gaps in the rapid nutritional risk screening methods for cancer patients. We aimed to evaluate the value of abridged scored patient-generated subjective global assessment (abPG-SGA) for nutritional risk screening and prognosis in cancer patients. The NRS 2002 and abPG-SGA scores of 100 malignant tumor patients hospitalized in our department in December 2020 were collected. Take NRS2002 ≥ 3 as the positive standard (risk of malnutrition). Data were analyzed using Kappa test, ROC curves, cut-off values and Kaplan–Meier. In the screening of 100 patients, 25.0% of patients were at risk of malnutrition (NRS2002), abPG-SGA yielded a sensitivity and specificity of 92.0% and 72.0%, respectively (area under curve [AUC] = 0.884, cut-off value ≥ 4.5); In the screening of patients with digestive system malignancies, 22.6% of patients were at risk of malnutrition (NRS2002), and the sensitivity and specificity of abPG-SGA were 91.67% and 87.80%, respectively (AUC = 0.945, cut-off value ≥ 5.5). The results of survival analysis showed that the overall survival (OS) of patients with abPG-SGA ≥ 5 and < 5, NRS2002 ≥ 3 and abPG-SGA < 5, NRS2002 < 3 and abPG-SGA ≥ 5 were significantly different (*P* < .0001), the OS of patients with NRS2002 ≥ 3 and abPG-SGA ≥ 5, NRS2002 < 3 and abPG-SGA < 5 were not significantly different (*P* > .05). Like NRS2002, abPG-SGA can also be used for malnutrition screening and prognosis judgment in cancer patients. It can quickly screen out cancer patients who may be at risk of malnutrition and facilitate the development of nutritional assessments.

## 1. Introduction

Cancer patients are a high-risk group of malnutrition. Studies have shown that the incidence of malnutrition in cancer patients ranges from 40% to 80%.^[1]^ Both American Society for Parenteral and Enteral Nutrition and European Society for Parenteral and Enteral Nutrition (ESPEN) recommend that all cancer patients should undergo nutritional risk screening and assessment.^[2,3]^ Nutritional Risk Screening 2002 (NRS2002) is the preferred nutritional risk screening method recommended by ESPEN and Chinese Society for Parenteral and Enteral Nutrition.^[3,4]^ Patient-Generated Subjective Global Assessment (PG-SGA) was developed on the basis of Subjective Global Assessment (SGA) first proposed by Ottery FD in 1994.^[5]^ It is an effective and specific nutritional assessment tool specially designed for cancer patients. It has been solemnly recommended and widely used by the American Dietitians Association (ADA).^[6–8]^ abridged scored Patient-Generated Subjective Global Assessment (abPG-SGA) is the first part of PG-SGA, which is completed by the patients. When screening tumor patients for nutritional risk using the NRS2002, if the patient is found to be at nutritional risk, the patient is further assessed using the PG-SGA, and based on the results of the assessment, the patient is intervened accordingly. If abPG-SGA can be used for nutritional risk screening of tumor patients, it will skip the NRS2002 step and go directly to parts B, C, and D of the PG-SGA, which not only saves the time spent on evaluating the NRS2002, but also eliminates the need to re-spend time on evaluating part A of the PG-SGA and improves the efficiency of clinical work. In this study, NRS2002 and the abPG-SGA were used to screen patients for nutritional risk in our department, in order to evaluate the feasibility of abPG-SGA in nutritional risk screening and the value of prognosis.

## 2. Object and method

### 2.1. Case selection and general information

Nutritional risk screening was performed on 186 patients with malignant tumors admitted to the oncology department of our hospital in December 2020. Inclusion criteria were adults (age ≥ 18 years) and had a nutritional evaluation by abPG-SGA and NRS2002 scale within 24 hours of admission. The patients were able-bodied and were expected to have a life expectancy of >3 months. Patients who cannot understand the content of the scale or cannot clearly express their meaning will be excluded. The study was approved by the ethics committee of our hospital. 100 patients were ultimately included. There were 59 males and 41 females. The age ranged from 30 to 87 years old, with a median age of 60 years old. There were 53 cases of digestive system tumors, 17 cases of respiratory system tumors, and 30 cases of other tumor types (breast cancer, cervical cancer, prostate cancer, etc).

### 2.2. Scoring method

NRS2002 scale is implemented according to ESPEN standards. Patients with a total score ≥ 3 are considered to be at risk with respect to nutritional. abPG-SGA is a patient self-assessment, the A score part in PG-SGA, excluding the medical staff assessment part. All nutritional scores were evaluated by trained nurses and clinical doctors.

### 2.3. Statistical methods

SPSS 25.0 software was used for data processing and analysis. Kappa test was used to analyze the consistency of abPG-SGA and NRS2002 screening methods (If the *P* value is <.05, it indicates that the 2 screening methods are consistent). The cut-off value was calculated by using the Youden index of ROC curve, and the sensitivity and specificity were further analyzed. Life-table estimation was performed using the Kaplan–Meier method to compare survival outcomes, and differences were evaluated using the log-rank test. A *P* value < .05 was considered statistically significant.

## 3. Results

### 3.1. Summary of malnutrition risk screening results of 2 screening methods

The specific malnutrition risk ratios are shown in Table 1. The risk ratio of malnutrition was 29.0% by the abPG-SGA and 25.0% by NRS 2002. There was consistency between the 2 methods (*P* = .000, Kappa test). In the survey of digestive system tumors, it was found that the malnutrition risk proportion with the NRS 2002 was 22.6% and that the proportion with the abPG-SGA was 32.1%. There was consistency between the 2 methods (*P* = .000, Kappa test). In the screening of respiratory system tumors, the proportion of malnutrition risk obtained by the 2 screening methods was the same (*P* = .036, Kappa test).

**Table 1:** Summary of malnutrition risk results for patients with various types of cancer.

Tumor site	N	Risk of malnutrition				Kappa test
		abPG-SGA		NRS2002		*P*
		n	%	n	%	
Digestive system	53	17	32.1%	12	22.6%	.000
Respiratory system	17	5	29.4%	5	29.4%	.036
Others	30	7	23.3%	8	26.7%	.000
Total	100	29	29%	25	25%	.000

abPG-SGA = abridged scored patient-generated subjective global assessment, NRS2002 = nutritional risk screening 2002.

### 3.2. The cut-off value was calculated according to the ROC curve

**Figure 1.**
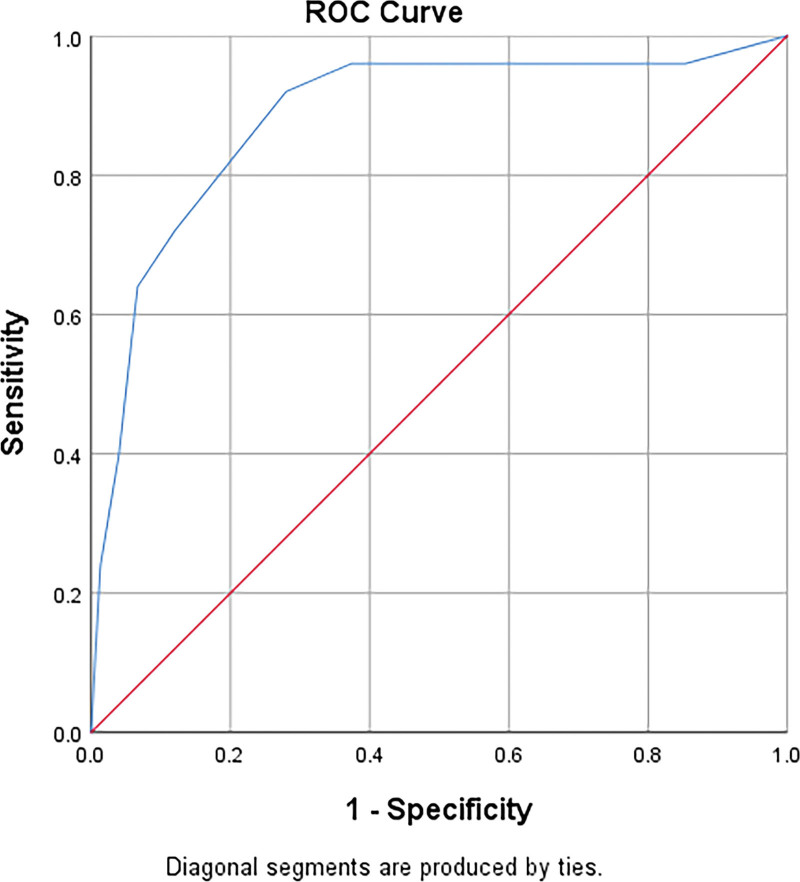
ROC curve of nutritional screening of 100 patients with malignant tumors. ROC curves = receiver operating characteristic curves.

**Figure 2.**
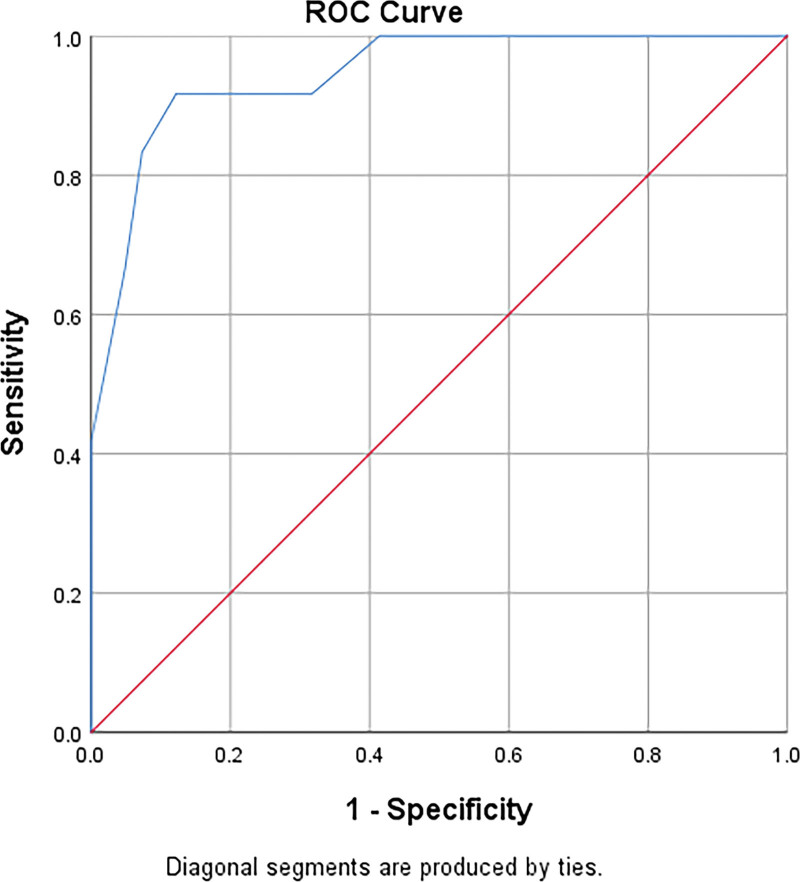
ROC nutritional curve screening for cancer patients. ROC = receiver operating characteristic.

The measured values of the abPG-SGA group were used as test variables, and those of the NRS 2002 group were used as reference variables. The area under curve (AUC) of 100 patients with malignant tumors was 0.884 (*P* < .05), with statistical significance, and the cut-off value was 4.5 (Table 2, Fig. 1); AUC of patients with digestive system malignant tumors was 0.945 (*P* < .05), with statistical significance, and the cut-off value was 5.5 (Table 2, Fig. 2).

**Table 2:** AUC and cut-off value of nutrition screening.

Group	Area	Std.Error	*P*	Asymptotic 95% confidence interval		Cutoff
				Lower bound	Upper bound	
100 patients with malignant tumors						
abPG-SGA	0.884	0.042	.000	0.801	0.967	4.5
Patients with digestive system tumors						
abPG-SGA	0.945	0.034	.000	0.878	1.000	5.5

abPG-SGA = abridged scored patient-generated subjective global assessment, AUC = area under curve.

### 3.3. Sensitivity and specificity

In the risk screening of malnutrition in 100 patients with malignant tumor, abPG-SGA score ≥ 5 was considered to be at risk of malnutrition (positive), with a sensitivity of 92.0% and specificity of 72.0% (Table 3). In the analysis of patients with digestive system malignant tumor, abPG-SGA score ≥ 6 was considered to be at risk of malnutrition (positive), and the sensitivity and specificity of abPG-SGA were 91.67% and 87.80%, respectively (Table 3).

**Table 3:** Sensitivity and specificity of nutritional screening.

Group		NRS2002		Sensitivity(%)	Specificity(%)
		Negative	Positive		
100 patients with malignant tumors					
abPG-SGA	Negative	54	2	92.00	72.00
	Positive	21	23		
Patients with digestive system cancer					
abPG-SGA	Negative	36	1	91.67	87.80
	Positive	5	11		

abPG-SGA = abridged scored patient-generated subjective global assessment, NRS2002 = nutritional risk screening 2002.

### 3.4. Survival analysis

**Figure 3.**
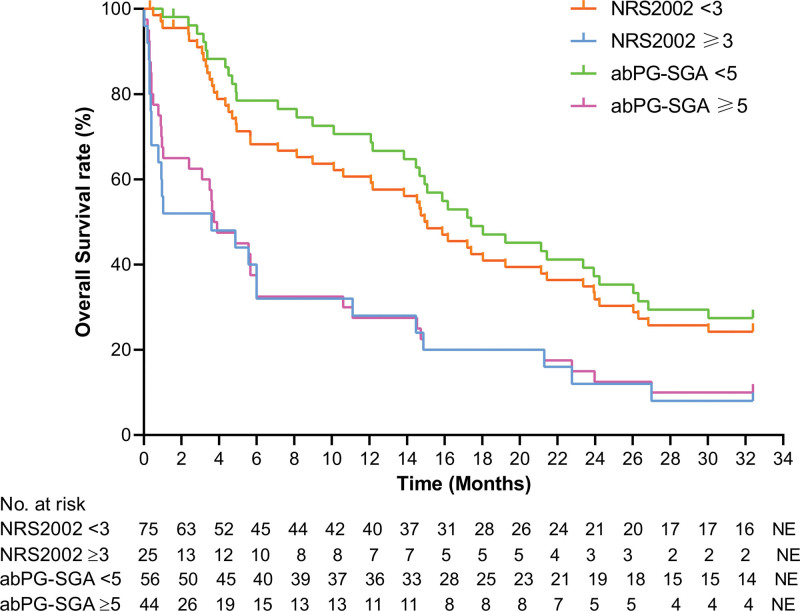
Kaplan–Meier OS curves for the 100 patients stratified as NRS2002 < 3, NRS2002 ≥ 3, abPG-SGA < 5 and abPG-SGA ≥ 5. abPG-SGA = abridged scored patient-generated subjective global assessment, NRS2002 = nutritional risk screening 2002, OS = overall survival.

The last follow-up time was July 31, 2023. Patients with NRS2002 ≥ 3 and abPG-SGA ≥ 5, NRS2002 < 3 and abPG-SGA < 5 were no statistical difference in overall survival (OS) (*P* > .05). The OS of patients with abPG-SGA ≥ 5 and < 5, NRS2002 ≥ 3 and abPG-SGA < 5, NRS2002 < 3 and abPG-SGA ≥ 5 were significantly different (*P* < .0001) (Fig. 3).

## 4. Discussion

American Society for Parenteral and Enteral Nutrition recommends that all cancer patients should undergo nutritional risk screening and nutritional assessment. NRS2002 is the preferred tool for nutritional risk screening of hospitalized patients.^[9,10]^ PG-SGA is mainly used for nutritional assessment of cancer patients.^[3,11]^ abPG-SGA is the short form of PG-SGA, which forgoes the physical exam, disease/condition, and metabolic considerations, but retains weight, eating, changes in activity and assessment of digestive tract symptoms.^[12–14]^ If abPG-SGA can be used for nutritional risk screening of tumor patients, part B, C and D can be directly evaluated on the basis of abPG-SGA during PG-SGA nutritional evaluation. In this way, there is no need to reevaluate part A of PG-SGA, and it saves the steps of nutritional risk screening with NRS2002, which is conducive to the development of nutritional screening.

In this study, nrs2002 ≥ 3 was regarded as the positive standard. Through analysis, it was found that there was consistency between the NRS2002 and abPG-SGA. The risk of malnutrition in 100 patients with malignant tumors was investigated by abPG-SGA, AUC was 0.884. AUC in patients with malignant tumor of digestive system was 0.945. All *p* values were < 0.05, indicating statistical significance. The AUC of the 2 sets of data was between 0.7–0.9, suggesting that the screening accuracy is high, that is, the abPG-SGA can be considered for the total population of cancer patients or the risk of malnutrition for malignant tumors of the digestive system. Among 100 patients, the results of the abPG-SGA screening method showed that the risk of malnutrition was 29.0%, and the cut-off value calculated according to the Youden index of the ROC curve was 4.5. When abPG-SGA score ≥ 5, ≥6, or ≥ 7 was classified as positive, the sensitivity was 92.0%, 72.0%, 64.0%, and the specificity was 72.0%, 88.0%, and 93.33%, respectively. The NRS2002 screening method showed that the risk of malnutrition was 25.0%. Subgroup analysis of respiratory system and digestive system showed that: In the screening of respiratory system tumors, the risk proportion of malnutrition obtained by the 2 screening methods was the same, while in the investigation of digestive system tumors, it was found that the risk proportion of malnutrition screened by NRS2002 method was 22.6%, and the risk proportion of malnutrition screened by abPG-SGA was 32.1%, when the score ≥ 6 was positive, the abPG-SGA yielded 91.67% sensitivity and 87.80% specificity, when the score ≥ 7 was positive, the sensitivity and specificity of abPG-SGA were 83.33% and 92.68%, respectively. In summary, with the same cut-off value, abPG-SGA has higher sensitivity than the general population when used for nutritional screening of malignant tumors of the digestive system. Gabrielson et al^[13]^ applied abPG-SGA to outpatient tumor patients, and took PG-SGA as the gold standard, and concluded that the sensitivity of abPG-SGA was 94% and the specificity was 78%. In this study, the sensitivity and specificity of positive abPG-SGA score ≥ 5 were basically consistent with the results of Gabrielson et al. We further compared the survival time of patients with NRS2002 score ≥ 3 and abPG-SGA score ≥ 5, NRS2002 score < 3 and abPG-SGA score < 5. It was found that there was no significant difference in OS between patients with NRS2002 score ≥ 3 and abPG-SGA score ≥ 5, and there was no significant difference in OS between patients with NRS2002 score < 3 and abPG-SGA score < 5. This shows that like NRS2002, abPG-SGA can also be used for nutritional risk screening and prognostic judgment. From the survival curve chart, we can see that the consistency of NRS2002 ≥ 3 and abPG-SGA ≥ 5 is higher than NRS2002 < 3 and abPG-SGA < 5.

Our research shows that abPG-SGA can be used for malnutrition screening of hospitalized patients with malignant tumors, especially for malnutrition risk screening of patients with gastrointestinal tumors. This can not only save the time used in this step of NRS2002, but also parts B, C and D of PG-SGA can be directly performed on the basis of abPG-SGA in subsequent nutritional assessment, which saves clinical working time and facilitates the development of nutritional assessment. This study also has some limitations, mainly limited to a small sample, single center study, and in the future, the sample size should be increased for analysis. Each type of malignant tumor has multiple other factors that affect the patient nutritional status, and more factors need to be combined for analysis. Some anthropometric indicators related to nutrition have not been collected, including hand grip strength, visual fat area, scale circulation, and accessory skeletal muscle mass index, which may more comprehensively reflect the specific nutritional status of patients. In addition, the study also lacks sufficient control over confounding factors, such as tumor staging and clinicopathological characteristics. Therefore, in future trial designs, it is important to collect as many confounding factors as possible to minimize the impact on the trial results. This study only analyzed nutritional screening for cancer patients and its relationship with OS, without stratified analysis of subsequent nutritional evaluation and intervention for patients, expecting later data.

## Author contributions

**Data curation:** Ying Zhang.

**Formal analysis:** Xiaoling Zhang, Ying Zhang.

**Investigation:** Ying Zhang, Yunyi Du, Qian Wu, Xiaoyu Wu, Xurong Li.

**Methodology:** Xiaoling Zhang.

**Supervision:** Wenqing Hu, Liang Zong.

**Writing – original draft:** Xiaoling Zhang, Ying Zhang.

**Writing – review & editing:** Jun Zhao.
